# Risk-Taking Behavior and the Consumption of Alcohol Mixed with Energy Drink among Australian, Dutch and UK Students

**DOI:** 10.3390/ijerph18105315

**Published:** 2021-05-17

**Authors:** Sean J. Johnson, Sarah Benson, Andrew Scholey, Chris Alford, Joris C. Verster

**Affiliations:** 1 Department of Health and Social Sciences, University of the West of England, Bristol BS16 1QY, UK; JohnsonS11@cardiff.ac.uk (S.J.J.); chris.alford@uwe.ac.uk (C.A.); 2 Centre for Trials Research, Cardiff University, Cardiff CF14 4YS, UK; 3 Centre for Human Psychopharmacology, Swinburne University, Melbourne, VIC 3122, Australia; sarahbenson@swin.edu.au (S.B.); andrew@scholeylab.com (A.S.); 4 Division of Pharmacology, Utrecht Institute for Pharmaceutical Sciences (UIPS), Utrecht University, 3584CG Utrecht, The Netherlands

**Keywords:** alcohol, energy drink, alcohol consumption, alcohol-related consequences, risk-taking, students, survey

## Abstract

The relationship between risk-taking behavior, alcohol consumption and negative alcohol-related consequences is well known. The current analyses were conducted to investigate whether alcohol mixed with energy drink (AMED) is related to risk-taking behavior and if there is a relationship between the amount of energy drink mixed with alcohol consumed, risk-taking behavior and negative alcohol-related consequences. Data from N = 1276 AMED consuming students from the Netherlands, UK and Australia who completed the same survey were evaluated. The analysis revealed that, compared to AMED occasions, on alcohol only (AO) occasions significantly more alcohol was consumed and significantly more negative alcohol-related consequences were reported. On both AO and AMED occasions, there was a strong and positive relationship between amount of alcohol consumed, level of risk-taking behavior and number of reported negative alcohol-related consequences. In contrast, the level of risk-taking behavior was not clearly related to energy drink consumption. Across risk-taking levels, differences in the amount of energy drink consumed on AMED occasions did not exceed one 250 mL serving of energy drink. When correcting for the amount of alcohol consumed, there were no statistically significant differences in the number of energy drinks consumed on AMED occasions between the risk-taking groups. In conclusion, alcohol consumption is clearly related to risk-taking behavior and experiencing negative alcohol-related consequences. In contrast, energy drink intake was not related to level of risk-taking behavior and only weakly related to the number of experienced negative alcohol-related consequences.

## 1. Introduction

Excessive alcohol consumption is a persistent, worldwide public health issue. Among 15–49-year olds, alcohol is the leading risk factor for premature mortality and disability, accounting for 10% of all deaths in this age group [1]. Acute alcohol consumption is partly responsible for this global harm via unintentional injuries, violence and suicide [2]. Consuming too much alcohol in a short space of time has been shown to impact brain functioning, including impaired activity in the pre-frontal cortex that is responsible for executive functions [3]. Acute alcohol consumption has been shown to affect central GABA_A_ receptors [4], disrupting the normal processes of the neocortex [5]. The overall effects include greater disinhibitory effects resulting in an increased propensity for risk-taking behaviors and alcohol-related harm. Indeed, scientific research has consistently shown that higher levels of alcohol consumption are associated with increased risk-taking behavior and more frequently experiencing negative alcohol-related consequences [6,7,8,9,10]. Some risky behaviors associated with alcohol-related consequences include driving under the influence of alcohol [11,12], drinking until blacking out [13], increased violence or aggression [14,15], sexual risk-taking [16] and other risks that may lead to injury [17]. Given the severity of consequences experienced, it is important to examine the factors that might mediate the relationship between heavy alcohol consumption and risk-taking behaviors.

One factor that has been suggested as influencing the relationship between alcohol and risk-taking is energy drinks [18,19]. Energy drinks are non-alcoholic beverages, typically containing 80 mg of caffeine per 250 mL serving size and other functional ingredients such as B-vitamins and taurine. It has been shown that individuals who consume alcohol mixed with energy drink (AMED) have higher risk-taking levels compared to individuals who do not consume AMED and they therefore may consume more alcohol and experience more alcohol-related negative consequences [20,21,22,23,24,25]. These comparisons do not, however, consider whether both heavier drinking and AMED consumption are a manifestation of some third variable [26]. Indeed, meta-analyses of within-subject comparisons (comparing AMED occasions with alcohol only (AO) occasions within the same subjects) show that the observed increased alcohol consumption is evident in both AMED and AO drinking occasions [26,27]. Similarly, meta-analyses show that individuals who consume AMED do not consume more alcohol on AMED occasions compared to AO occasions [26,27,28]. In addition, compared to AO drinking occasions, significantly fewer negative consequences were reported for AMED occasions [23,24].

Notwithstanding these observations, it is of interest to further investigate the possible interplay between energy drink and alcohol consumption and its relationship with risk-taking and experiencing alcohol-related negative consequences [19,28]. Previously, Verster et al. [28] concluded that “The literature is overwhelmingly consistent with the notion that AMED consumption is just one manifestation of an underlying trait for greater alcohol consumption along with a cluster of other risky behaviors”. Consistent with this literature, we hypothesized that mixing alcohol with energy drinks has no relevant influence on alcohol consumption levels and subsequent negative alcohol-related consequences. We also hypothesized that there would be a direct positive relationship between the amount of alcohol consumed and the level of risk-taking behavior and number of experienced alcohol-related negative consequences, independent of energy drink consumption. The aim of the current study was to further evaluate these hypotheses using large student samples drawn from three countries. Students were recruited as participants because this group comprises regular consumers of AMED [26,28]. The study was conducted in three different countries. This was done in order to verify whether the hypotheses could be confirmed, irrespective of possible different drinking cultures in different countries. Australia, UK and the Netherlands were chosen as in each of these countries both alcohol and energy drink consumption are popular.

## 2. Materials and Methods

Data were evaluated from three directly comparable online surveys conducted among Dutch, UK and Australian students [29,30,31]. The survey was designed to investigate the possible impact of mixing alcoholic beverages with energy drinks on overall alcohol consumption and alcohol-related consequences. The survey was completed online via SurveyMonkey^®^ (Palo Alto, CA, USA) in Dutch language in the Netherlands and English language in the UK and Australia. The studies were conducted according to the guidelines of the Declaration of Helsinki. The Dutch survey was reviewed by The Medical Ethical Review Board Twente, but no formal medical ethics approval was required. The UK survey was approved by the University of the West of England Faculty Ethics committee (approval number: HAS/14/03/57) and the Australian survey was approved by the Swinburne University Human Research Ethics Committee (Reference 2012/045). Informed consent was obtained from all participants involved in the studies. Detailed information on the content and design of the survey [32] and a comparison between the countries on alcohol intake [33] have been published elsewhere.

For the current evaluation we investigated the relationship between alcohol and energy drink consumption, risk-taking behavior and negative alcohol-related consequences. These assessments are described in the next sections. Students in the age of 18 to 30 years old who consumed AMED during the past month were included. There were no exclusion criteria.

### 2.1. Alcohol and Energy Drink Consumption

Alcohol consumption questions were adapted from the Quick Drinking Screen [34,35] to assess beverage consumption during the past 30 days. The questions comprised past month’s number of alcoholic drinks consumed on a typical (‘usual’) drinking occasion and on past month’s heaviest drinking occasion. Guidance was provided regarding the standardized size of alcoholic drinks using pictures of different serving sizes (e.g., glass, shot, bottle) along with the content in ml, and how to transfer common amounts (e.g., a bottle of wine) into standard units (of 10 g alcohol in each country). The questions were answered for both AMED and AO drinking occasions. For AMED occasions, it was also assessed how many energy drinks (1 unit was defined as a standard serving size of 250 mL) were mixed with alcohol on the usual and past month’s heaviest drinking occasion. AMED consumption was defined as consuming an energy drink within +/−2 h of alcohol consumption, which represents a conservative definition of ‘mixing’ [32].

### 2.2. Level of Risk Taking Behavior

The risktaking-18 items (RT-18) questionnaire [36] was completed to assess the participants’ level of risk-taking behavior. The 18 items can be answered with ‘no’ or ‘yes’ (0 or 1 point, depending on the item) and the sum score of the RT-18 ranges from 0 (no risk-taking) to 18 (extreme risk-taking). Based on previous research [36] and the distribution of the current data, participants were allocated to having a low risk-taking profile (RT-18 score ≤ 5), moderate risk-taking profile (RT-18 score from 6 to 12) or high risk-taking profile (RT-18 score ≥ 13).

### 2.3. Negative Alcohol-Related Consequences

Separate for both AMED and AO drinking occasions, participants completed the Brief Young Adult Alcohol Consequences Questionnaire (BYAACQ) [37,38]. The BYAACQ consists of 24 items representing negative consequences of alcohol consumption. The items could be answered with ‘yes’ or ‘no’, depending on whether or not the participant experienced the negative consequence within the past year while drinking AMED and while drinking AO. Based on previous research [38] and the distribution of the current data, participants were allocated to having a low number of negative alcohol-related consequences (BYAACQ score ≤ 4), having a moderate number of negative alcohol-related consequences (BYAACQ score of 5–8) or having a high number of negative alcohol-related consequences (BYAACQ score ≥ 9).

### 2.4. Statistical Analysis

Data were analyzed using IBM SPSS Version 27 (IBM Corp., Armonk, NY, USA). For the current analysis, participants were included if they were students between the age of 18 and 30 years old and consumed AMED during the past month. Participants with missing data were excluded from the analysis. Moreover, data on energy drink consumption from three UK participants that reported unreliably high numbers of energy drinks on their heaviest drinking occasion (≥30 250 mL energy drinks) were also omitted from the analysis. The mean and standard deviation (SD) were computed for all variables and the distribution of the data was checked for normality. Alcohol and energy drink consumption was compared between the levels of risk-taking behavior and between the levels of experienced negative alcohol-related consequences. These comparisons were performed for the sample as a whole and for the individual countries. As the data were not normally distributed, the Independent-Samples Kruskal–Wallis test was used. If the main effect was statistically significant, post-hoc pairwise comparisons were conducted, applying appropriate Bonferroni correction to account for multiple comparisons. Differences were considered statistically significant if *p* < 0.05. Sex differences were evaluated with the Independent-Samples Mann-Whitney U test. Differences between men and women were considered statistically significant if *p* < 0.05.

## 3. Results

Of the N = 2205 AMED consumers who started the survey, N = 1267 completed all assessments. The sample under evaluation comprised 1267 AMED consumers (N = 553 men and N = 714 women). Their demographics are summarized in Table 1.

In each country, significantly more women than men completed the survey (*p* < 0.0001 for Netherlands and Australia, *p* < 0.02 for UK). Dutch participants were significantly younger (*p* < 0.0001) and started consuming alcohol regularly at a younger age compared to UK (*p* < 0.0001) and Australian students (*p* < 0.0001). Risk-taking scores and reported negative alcohol-related consequences were significantly lower in the Netherlands compared to the UK and Australian sample (*p* < 0.0001 for each pairwise comparison).

**Figure 1:**
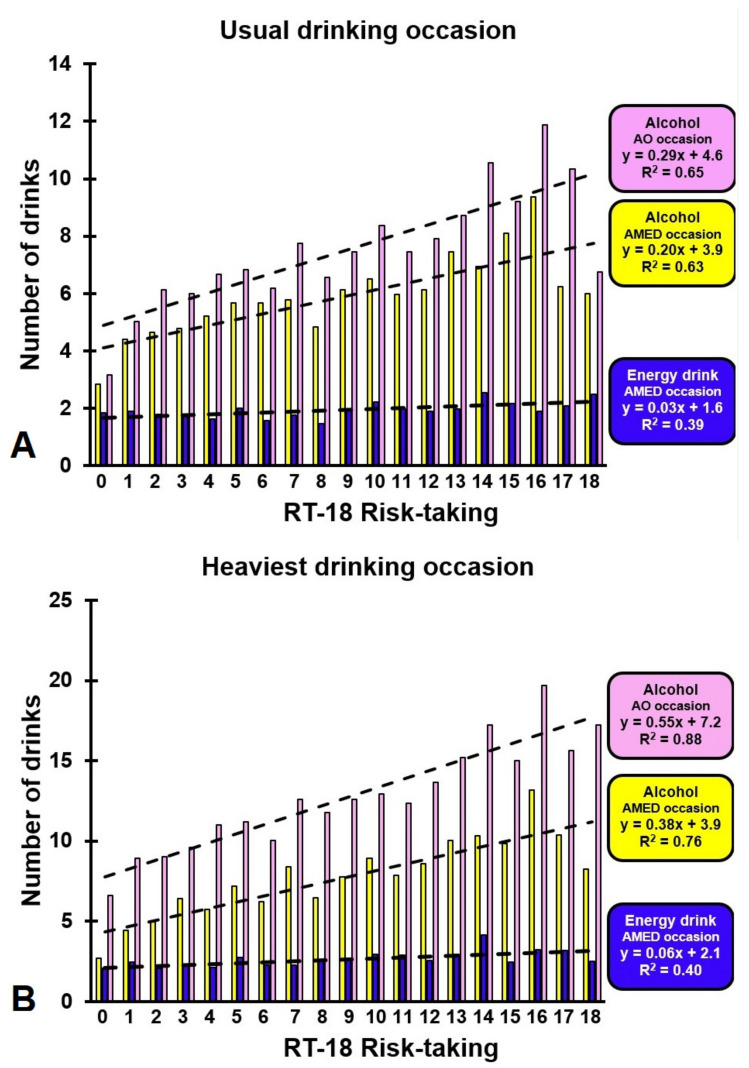
The relationship between risk-taking and alcohol and energy drink consumption. Shown are the mean number of drinks consumed for each RT-18 risk-taking score for usual drinking occasions (**A**) and past month heaviest drinking occasion (**B**). Abbreviations: AO = alcohol only; AMED = alcohol mixed with energy drink; RT-18 = risk-taking questionnaire, 18 items.

Figure 1 illustrates the relationship between risk-taking and alcohol and energy drink consumption. The trendlines in Figure 1 suggest that for both usual and the past month’s heaviest AO and AMED occasions there is a clear relationship between alcohol intake and level of risk-taking behavior, whereas this relationship is not seen between risk-taking behavior and the number of energy drinks consumed on AMED occasions.

Table 2 summarized alcohol intake according to risk-taking behavior level. Table 3 summarized the number of energy drinks consumed on AMED occasions according to risk-taking behavior level. 

Table 2 summarizes the number of alcoholic drinks consumed on AMED and AO occasions (usual and past month heaviest drinking occasion) for the three countries and the three risk-taking levels. Within-subject comparisons revealed that, compared to AMED occasions, participants consumed significantly more alcohol on both usual AO occasions (*p* < 0.0001) and past month heaviest AO drinking occasions (*p* < 0.0001). This observation was consistent for usual drinking occasions across the three countries (*p* < 0.0001 for the Netherlands and UK; *p* = 0.001 for Australia) and across risk-taking groups (all comparisons *p* < 0.0001). The observation was also consistent for past month heaviest drinking occasions across the three countries (all comparisons *p* < 0.0001) and across risk-taking groups (all comparisons *p* < 0.0001). 

It is also evident from Table 2 that with higher levels of risk-taking the reported number of alcoholic drinks consumed was higher, both on AO and AMED occasions, and both on usual drinking occasions and past month heaviest drinking occasion. Table 3 shows that the differences in number of energy drinks consumed on AMED occasions between the risk-taking groups are small, i.e., ≤1 serving of 250 mL energy drink. When correcting for the number of alcoholic drinks consumed, the number of energy drinks consumed on AMED occasions did not significantly differ between any of the risk-taking groups.

Table 4 summarizes alcohol consumption for the three BYAACQ groups. Table 4 shows that, consistent across countries and observed for both AO and AMED occasions, increasing numbers of negative alcohol-related consequences are associated with higher levels of alcohol intake.

Table 5 summarized the relationship between the number of energy drinks consumed on AMED occasions and reported negative alcohol-related consequences. The analysis revealed that participants who reported more negative alcohol-related consequences consumed more energy drinks on AMED occasions. However, the absolute differences in number of energy drinks consumed between the groups was small and except for the UK equaled less than one 250 mL serving of energy drink between the lowest and highest BYAACQ groups. Of interest, energy drink intake of the moderate BYAACQ group of the Dutch and Australian sample was higher compared to the highest BYAACQ group.

### Sex Differences

To evaluate possible sex differences we analyzed the combined dataset, comparing outcomes of 553 male and 714 female participants. Women started consuming alcohol regularly at a later age then men and consume significantly less alcohol (both quantity and frequency) on AMED and AO occasions compared to men (See Table 6). The data show that on both AO and AMED occasions women consumed significantly less alcohol than men. Women also consumed significantly fewer energy drinks on AMED occasions. Further, women had significantly lower risk-taking scores and reported significantly fewer negative alcohol-related consequences for both AO and AMED occasions. No sex differences were found for age or age of regular alcohol consumption.

Table 7 summarizes alcohol consumption for the three risk-taking groups for men and women. Among both men and women, the number of alcoholic drinks was significantly higher at subsequent levels of risk-taking. For each comparison at each risk-taking level, men consumed significantly more alcohol than women (*p* < 0.05). Table 8 summarizes energy drink consumption for the three risk-taking groups for men and women. While energy drink intake increased at higher risk-taking levels for both men and women, except for the difference in energy drink consumption between the lowest and highest risk-taking level of men on their heaviest drinking occasion (1.3 servings, *p* = 0.010), the magnitude of the all other differences in energy drink consumption between the risk-taking levels was always less than one can of energy drink and not statistically significant. Taken together, among both men and women a clear relationship between the level of risk-taking and alcohol consumption was observed, whereas the differences in energy drink consumption were usually not significant between the risk-taking levels. 

Table 9 summarizes alcohol consumption for the three BYAACQ groups for men and women. Among both men and women, the number of alcoholic drinks was significantly higher at subsequent BYAACQ levels. For each comparison at each risk-taking level, men consumed significantly more alcohol than women (*p* < 0.05). Table 10 summarizes energy drink consumption for the BYAACQ groups for men and women. While energy drink intake increased at higher BYAACQ levels for both men and women, the absolute differences were small and less than one can of energy drink. For usual AMED occasions, the difference in energy drink consumption between the lowest and highest BYAACQ level was significant in both men (0.9 servings, *p* < 0.0001) and women (0.5 servings, *p* = 0.023) and in men only between the medium and highest BYAAC level (0.6 servings, *p* = 0.002) were statistically significant. For the heaviest drinking occasion, post-hoc analysis revealed significant differences between energy drink consumption between the lowest and highest BYAAC level for both men (1.2 servings, *p* < 0.0001) and women (0.6 serving, *p* = 0.016). All other differences in energy drink consumption between the BYAACQ levels were not statistically significant. Taken together, among both men and women a clear relationship between the level of experienced negative alcohol-related consequences and alcohol consumption were observed. In contrast, differences in energy drink consumption between the BYAACQ levels were usually less than one can of energy drink. 

## 4. Discussion

The aim of the current study was to investigate energy drink and alcohol consumption in the context of risk-taking and experiences of alcohol-related negative consequences. Across the three countries studied, it was found that significantly less alcohol is consumed on AMED occasions compared with AO occasions (see Table 2). Consistent with this finding, significantly fewer negative alcohol-related consequences are reported for AMED occasions compared to AO occasions (see Table 4). 

There are, however, differences between the countries in consumption levels, which have been discussed elsewhere [33]. For example in general the UK sample consumes more alcohol than the Dutch and Australian sample. However, the differences in alcohol consumption between AO and AMED occasions were consistent between countries and across risk-taking levels. Significantly less alcohol is consumed on AMED occasions than AO occasions. These findings are in line with previous research [27,28] showing that AMED consumers in general drink more alcohol than AO consumers. However, using a within-subject design comparing AMED with AO occasions, decreased alcohol consumption on AMED occasions has been consistently found in studies around the world. The results suggests that low, medium or high levels of alcohol consumption depend on one’s personality characteristics and are not related to the chosen non-alcoholic mixer. 

The current analysis revealed that an increased level of risk-taking behavior was associated with increased alcohol intake and that energy drink consumption did not modify this. Specifically, this association was seen for both AMED and AO occasions and was more pronounced in the latter. This study adds to the literature that the level of risk-taking behavior was not clearly related to energy drink consumption. Across risk-taking levels, variation in the amount of energy drink consumed on AMED occasions was no more than a single 250 mL serving of energy drink for each country. When correcting for amount of alcohol consumed, there were no statistically significant differences in the number of energy drinks consumed on AMED occasions between the risk-taking groups. Thus, whereas the level of risk-taking behavior is unrelated to the amount of energy drink consumed on AMED occasions, the risk-taking level is associated with the overall amount of alcohol consumed, both on AO and AMED occasions. The data thus support our hypothesis that mixing alcohol with energy drinks has no relevant influence on alcohol consumption levels and subsequent negative alcohol-related consequences. Across countries it was consistently found that the relationship with risk-taking is driven by the amount of alcohol consumed and not by the amount of energy drinks consumed on AMED occasions. In contrast to the large differences observed for alcohol, the amount of energy drink consumed usually differed less than one 250 mL can between the risk-taking of BYAACQ levels. This was observed in both men and women. 

Regarding reported alcohol-related negative consequences, there was also a strong association between the amount of alcohol consumed and the number of experienced past year negative alcohol-related consequences. With greater alcohol intake, either on AO or AMED occasions, more negative alcohol-related consequences were reported. The association between the number of energy drinks mixed with alcohol and negative alcohol-related consequences were also statistically significant, but was less pronounced compared to the association with between alcohol intake and consequences. It is important to note that the observed difference in number of energy drinks consumed on AMED occasions between the lowest and highest BYAACQ groups was about one 250 mL serving of energy drink, which is much smaller than the corresponding increase of 3 to 4 alcoholic drinks (see Figure 1). Finally, as the chances of mixing energy drink with alcohol becomes larger when more alcohol is consumed, it is understandable that the total amount of alcohol consumed influences the relationship between energy drink intake and consequences.

The current findings are in line with previous research showing that higher levels of alcohol consumption are associated with increased risk-taking behavior and more frequently experiencing negative alcohol-related consequences [6,7,8,9,10]. The findings are also in agreement with previous meta-analyses showing that individuals who consume AMED do not consume more alcohol on AMED occasions compared to AO occasions [26,27,28]. Finally, the data confirm previous studies that conducted within-subject comparisons showing that, compared to AO occasions, consuming AMED does not increase negative alcohol-related consequences or risk-taking behavior [23,24].

The analysis revealed several sex differences. Men reported consuming significantly more alcohol than women, and more frequently. This was found for both AO and AMED occasions. In line, men reported significantly higher levels of risk-taking behavior compared to women and significantly more negative alcohol-related consequences. Among both men and women clear relationships between the level of risk-taking and alcohol consumption and between the level of experienced negative alcohol-related consequences and alcohol consumption were observed, whereas the differences in energy drink consumption were usually not significantly related to risk-taking levels or the number of reported negative alcohol-related consequences. Finally, in both men and women alcohol consumption on AMED occasions was significantly lower compared to the amount of alcohol consume on AO occasions. 

A strength of the current study is that the same survey was replicated in three countries. Validated questionnaires were used to assess risk-taking and negative alcohol-related consequences. The key research findings are consistent across the three countries, which strengthens the belief that the study outcomes can be generalized to other countries. While small differences in statistical significances were observed between the countries, the relationship between alcohol intake, risk-taking and negative consequences was evident in all three countries [33], whereas risk-taking was unrelated to the number of energy drinks consumed on AMED occasions. 

A limitation of the current study is that the data were collected retrospectively. Therefore, recall bias may have influenced the study outcomes. Future prospective studies should therefore be conducted before these findings can be confirmed. Secondly, the samples under investigation comprised only students, in the age range of 18 to 30 years old. It is therefore unclear to what extend our findings can be generalized to other age groups and non-student populations. This is an important area for future research, as a recent comparison of student and non-student populations revealed that non-students consistently consume more alcohol and are involved in a greater number of negative alcohol-related consequences than students [31]. Third, in the surveys we assessed negative alcohol-related consequences. However, the items of the BYAACQ could be answered only with ‘yes’ or ‘no’, and therefore we were unable to assess the frequency of occurrence of these consequences. As there are significantly more AO occasions than AMED occasions, this may have an impact on the frequency of experiencing negative consequences. On the other hand, the impact of the latter may be relatively small, as a study that matched the frequency of AMED and AO occasions also reported lower odds of experiencing negative alcohol-related consequences on AMED occasions compared to AO occasions [24].

## 5. Conclusions

The data suggest that the level of risk-taking behavior is associated with the amount of alcohol consumed. In contrast, level of risk-taking behavior seems unrelated to the amount of energy drink consumed on AMED occasions. The experienced negative alcohol-related consequences associated with the drinking occasion are clearly related to the amount of alcohol consumed, whereas the amount of energy drink consumed had relatively limited impact. The data show that alcohol consumption levels among young adults are relatively high, and this has implications for both future research and policy makers. The current research suggest that they should focus on the negative effects of excessive consumption of alcohol per se. Focus on non-alcoholic mixers such as energy drinks does not help to limit overall alcohol consumption, nor does it reduce alcohol-related risk-taking or negative consequences.

## Figures and Tables

**Table 1:** Demographics and study outcomes.

Demographics	Overall	The Netherlands	UK	Australia
N	1267	713	441	113
Male/female ratio	553/714	306/407	209/232	38/75
Age (years)	21.1 (2.3)	21.4 (2.3)	20.5 (2.0) Υ	20.9 (2.7) ^†^
Age of regular alcohol use	16.7 (1.7)	16.5 (1.7)	16.9 (1.7) Υ	17.1 (1.9) ^†^
RT-18 total risk-taking score	8.3 (4.1)	7.5 (4.1)	9.0 (4.1) Υ	10.4 (3.3) ^† ‡^
BYAACQ AO	7.2 (5.0)	5.4 (4.0)	9.5 (5.4) Υ	9.1 (4.6) ^†^
BYAACQ AMED	4.9 (4.8)	3.1 (3.6)	7.6 (5.1) Υ	6.5 (4.3) ^†^

Mean and standard deviation (SD, between brackets) are shown. Significant differences, after Bonferroni’s correction for multiple comparisons, between the countries are indicated as follows: The Netherlands and UK by Υ, between the Netherlands and Australia by ^†^ and between UK and Australia by ^‡^. Abbreviations: RT-18 = risk-taking questionnaire, 18 items; BYAACQ = brief young adult alcohol consequences questionnaire; AO = alcohol only; AMED = alcohol mixed with energy drink.

**Table 2:** Alcohol consumption and risk-taking.

	Number of Alcoholic Drinks			Overall	Pairwise Comparisons		
RT-18 Risk Taking Group	Low (0–5)	Moderate (6–12)	High (13–18)	*p*-Value	Low vs. Moderate	Moderate vs. High	Low vs. High
**Usual AO occasion**							
Overall	6.1 (4.5)	7.4 (4.8)	9.8 (6.1)	<0.0001 *	<0.0001 *	<0.0001 *	<0.0001 *
The Netherlands	5.3 (3.8)	6.6 (3.9)	8.1 (4.5)	<0.0001 *	<0.0001 *	<0.0001 *	0.002 *
UK	8.3 (5.6)	9.2 (5.7)	12.3 (7.2)	<0.0001 *	0.215	0.001 *	<0.0001 *
Australia	4.8 (1.9)	5.8 (3.2)	7.6 (4.1)	0.053	-	-	-
**Past month heaviest AO occasion**							
Overall	10.1 (6.3)	12.3 (6.9)	16.4 (8.7)	<0.0001 *	<0.0001 *	<0.0001 *	<0.0001 *
The Netherlands	9.5 (5.6)	11.9 (6.2)	15.6 (7.9)	<0.0001 *	<0.0001 *	<0.0001 *	<0.0001 *
UK	11.9 (7.5)	13.9 (8.0)	18.1 (9.3)	<0.0001 *	0.078	<0.0001 *	<0.0001 *
Australia	6.5 (3.7)	9.2 (4.5)	13.3 (8.0)	0.007 *	0.354	0.045 *	0.016 *
**Usual AMED occasion**							
Overall	5.0 (3.4)	5.9 (3.9)	7.6 (4.8)	<0.0001 *	<0.0001 *	<0.0001 *	<0.0001 *
The Netherlands	4.8 (3.1)	6.0 (3.9)	7.7 (4.5)	<0.0001 *	0.001 *	0.001 *	<0.0001 *
UK	5.5 (4.2)	6.0 (4.3)	8.0 (5.2)	0.001 *	0.839	0.005 *	0.001 *
Australia	3.8 (2.0)	4.9 (2.9)	6.2 (4.0)	0.166	-	-	-
**Past month heaviest AMED occasion**							
Overall	5.8 (5.1)	7.8 (6.2)	10.5 (8.6)	<0.0001 *	<0.0001 *	<0.0001 *	<0.0001 *
The Netherlands	5.2 (4.2)	7.4 (5.8)	9.4 (7.8)	<0.0001 *	<0.0001 *	0.127	<0.0001 *
UK	7.2 (6.9)	8.6 (7.1)	11.7 (8.9)	<0.0001 *	0.132	0.003 *	<0.0001 *
Australia	5.8 (3.4)	6.9 (4.7)	10.6 (9.8)	0.378	-	-	-

Mean and standard deviation (SD, between brackets) number of alcoholic drinks are shown. Post-hoc pairwise comparisons were conducted with Bonferroni’s correction. * = Differences are significant if *p* < 0.05. - = no post-hoc tests conducted as main effect was not significant. Abbreviations: AO = alcohol only; AMED = alcohol mixed with energy drink.

**Table 3:** Energy drinks mixed with alcohol consumption and risk-taking.

	Number of Energy Drinks on AMED Occasion			Overall	
RT-18 Risk Taking Group	Low (0–5)	Moderate (6–12)	High (13–18)	*p*-Value	Corrected *p*-Value ^1^
**Usual AMED Occasion**					
Overall	1.8 (1.3)	1.8 (1.5)	2.2 (1.9)	0.011 *	0.457
The Netherlands	1.7 (1.3)	1.6 (1.0)	1.9 (1.8)	0.464	0.073
UK	2.1 (1.3)	2.3 (2.0)	2.6 (2.2)	0.219	0.744
Australia	2.1 (1.4)	1.7 (1.4)	1.7 (0.8)	0.171	0.271
**Past Month Heaviest AMED Occasion**					
Overall	2.3 (1.5)	2.6 (2.2)	3.2 (2.8)	0.001 *	0.053
The Netherlands	2.2 (1.5)	2.3 (2.0)	3.0 (2.9)	0.118	0.069
UK	2.7 (1.6)	3.0 (2.2)	3.7 (3.0)	0.016 *	0.128
Australia	2.0 (0.8)	2.5 (2.6)	2.1 (1.1)	0.798	0.315

Mean and standard deviation (SD, between brackets) number of energy drinks consumed on AMED occasions are shown. Post-hoc pairwise comparisons were conducted with Bonferroni’s correction. * = Differences are significant if *p* < 0.05. ^1^ = *p*-value corrected for the number of alcoholic drinks consumed. Abbreviations: AMED = alcohol mixed with energy drink.

**Table 4:** Alcohol consumption and negative alcohol-related consequences.

	Number of Alcoholic Drinks			Overall	Pairwise Comparisons		
BYAACQ Group	Low (0–4)	Moderate (5–8)	High (9–24)	*p*-Value	Low vs. Moderate	Moderate vs. High	Low vs. High
**Usual AO Occasion**							
Overall	5.4 (3.5)	7.1 (4.5)	9.9 (5.9)	<0.0001 *	<0.0001 *	<0.0001 *	<0.0001 *
The Netherlands	5.0 (3.2)	6.8 (4.3)	9.0 (4.3)	<0.0001 *	<0.0001 *	<0.0001 *	<0.0001 *
UK	6.9 (4.5)	8.5 (4.8)	11.2 (6.8)	<0.0001 *	0.042 *	0.001 *	<0.0001 *
Australia	5.1 (2.5)	5.2 (3.6)	7.1 (3.3)	0.001 *	1.000	0.001 *	0.060
**Past Month Heaviest AO Occasion**							
Overall	9.0 (5.4)	12.3 (6.6)	16.0 (8.0)	<0.0001 *	<0.0001 *	<0.0001 *	<0.0001 *
The Netherlands	8.8 (5.1)	12.5 (5.7)	17.0 (7.2)	<0.0001 *	<0.0001 *	<0.0001 *	<0.0001 *
UK	10.2 (6.5)	13.4 (8.1)	16.2 (8.6)	<0.0001 *	0.018 *	0.006 *	<0.0001 *
Australia	7.0 (3.9)	8.2 (4.4)	12.5 (6.4)	<0.0001 *	1.000	<0.0001 *	0.001 *
**Usual AMED Occasion**							
Overall	4.9 (3.3)	7.1 (4.5)	7.4 (4.6)	<0.0001 *	< 0.0001 *	<0.0001 *	<0.0001 *
The Netherlands	5.1 (3.3)	8.5 (4.6)	7.7 (4.2)	<0.0001 *	<0.0001 *	1.000	<0.0001 *
UK	4.7 (3.7)	6.5 (4.5)	7.5 (4.9)	<0.0001 *	0.002 *	0.227	<0.0001 *
Australia	4.2 (2.5)	5.3 (2.7)	6.3 (4.0)	0.024 *	0.222	1.000	0.028 *
**Past Month Heaviest AMED Occasion**							
Overall	6.1 (5.2)	10.0 (8.0)	10.0 (7.3)	<0.0001 *	<0.0001 *	<0.0001 *	<0.0001 *
The Netherlands	5.8 (4.8)	10.5 (6.9)	11.0 (7.6)	<0.0001 *	<0.0001 *	1.000	<0.0001 *
UK	6.8 (6.3)	10.4 (9.3)	9.7 (6.9)	<0.0001 *	<0.0001 *	1.000	<0.0001 *
Australia	6.6 (5.4)	7.1 (4.6)	9.9 (8.7)	0.255	-	-	-

Mean and SD (between brackets) number of alcoholic drinks are shown. Post-hoc pairwise comparisons were conducted with Bonferroni’s correction. * = Differences are significant if *p* < 0.05. - = no post-hoc tests conducted as main effect was not significant. Abbreviations: BYAACQ = brief young adult alcohol consequences questionnaire; AO = alcohol only; AMED = alcohol mixed with energy drink.

**Table 5:** Energy drinks mixed with alcohol consumption and negative consequences.

	Number of Energy Drinks on AMED Occasion			Overall	Pairwise Comparisons			
BYAACQ Group	Low (0–4)	Moderate (5–8)	High (9–24)	*p*-Value	Low vs. Moderate	Moderate vs. High	Low vs. High	Corrected *p*-Value ^1^
**Usual AMED Occasion**								
Overall	1.6 (1.2)	2.1 (1.8)	2.4 (1.9)	<0.0001 *	<0.0001 *	0.012 *	<0.0001 *	<0.0001 *
The Netherlands	1.6 (1.1)	2.0 (1.7)	1.6 (1.0)	0.119	-	-	-	0.027 *
UK	1.8 (1.2)	2.2 (2.0)	2.8 (2.2)	<0.0001 *	0.459	0.001 *	<0.0001 *	0.014 *
Australia	1.6 (1.2)	2.0 (1.8)	1.6 (0.9)	0.239	-	-	-	0.135
**Past Month Heaviest AMED Occasion**								
Overall	2.2 (1.8)	3.0 (2.5)	3.3 (2.5)	<0.0001 *	<0.0001 *	0.347	<0.0001 *	<0.0001 *
The Netherlands	2.2 (1.8)	3.1 (2.9)	2.7 (1.8)	<0.0001 *	<0.0001 *	1.000	0.018 *	0.093
UK	2.4 (1.8)	3.0 (1.9)	3.7 (2.7)	<0.0001 *	0.004 *	0.120	<0.0001 *	<0.0001 *
Australia	2.0 (1.6)	2.8 (3.2)	2.4 (1.9)	0.239	-	-	-	0.365

Mean and SD (between brackets) number of energy drinks consumed on AMED occasions are shown. Post-hoc pairwise comparisons were conducted with Bonferroni’s correction. - = no post-hoc tests conducted as main effect was not significant. * = Differences are significant if *p* < 0.05. ^1^ = *p*-value corrected for the number of alcoholic drinks consumed. Abbreviations: BYAACQ = brief young adult alcohol consequences questionnaire; AMED = alcohol mixed with energy drink.

**Table 6:** Sex differences.

Demographics and Assessments	Men (N = 553)	Women (N = 714)	*p*-Value
Age (years)	21.0 (2.3)	21.1 (2.3)	0.941
Age of regular alcohol use	16.6 (1.7)	16.8 (1.7)	0.247
RT-18 total risk-taking score	9.1 (4.0)	7.7 (4.2)	<0.0001 *
AO occasions			
Number of alcoholic drinks (usual occasion)	9.3 (5.9)	6.0 (3.8)	<0.0001 *
Number of alcoholic drinks (heaviest occasion)	15.6 (8.0)	9.9 (5.6)	<0.0001 *
BYAACQ (AO occasions)	8.3 (5.2)	6.4 (4.7)	<0.0001 *
AMED occasions			
Number of alcoholic drinks (usual occasion)	7.3 (4.7)	4.8 (3.0)	<0.0001 *
Number of energy drinks (usual occasion)	2.1 (1.8)	1.7 (1.2)	<0.0001 *
Number of alcoholic drinks (heaviest occasion)	9.8 (7.6)	6.1 (5.2)	<0.0001 *
Number of energy drinks (heaviest occasion)	3.1 (2.6)	2.3 (1.7)	<0.0001 *
BYAACQ (AMED occasions)	5.9 (5.2)	4.2 (4.3)	<0.0001 *

Mean and standard deviation (SD, between brackets) are shown. Significant differences (*p* < 0.05) between men and women are indicated by *. Abbreviations: BYAACQ = brief young adult alcohol consequences questionnaire; AO = alcohol only; AMED = alcohol mixed with energy drink.

**Table 7:** Alcohol consumption and risk-taking.

	Number of Alcoholic Drinks			Overall	Pairwise Comparisons		
RT-18 Risk Taking Group	Low (0–5)	Moderate (6–12)	High (13–18)	*p*-Value	Low vs. Moderate	Moderate vs. High	Low vs. High
**Usual AO Occasion**							
Men	7.6 (5.7)	9.0 (5.3)	11.6 (7.0)	<0.0001 *	0.003 *	0.001 *	<0.0001 *
Women	5.4 (3.6)	6.0 (3.7)	7.6 (3.9)	<0.0001 *	0.017 *	<0.0001 *	<0.0001 *
**Past month heaviest AO Occasion**							
Men	13.0 (7.1)	15.3 (7.5)	18.8 (9.2)	<0.0001 *	0.009 *	0.002 *	<0.0001 *
Women	8.5 (5.3)	9.8 (5.1)	13.4 (6.9)	<0.0001 *	0.003 *	<0.0001 *	<0.0001 *
**Usual AMED Occasion**							
Men	6.5 (4.2)	7.2 (4.5)	8.5 (5.5)	0.014 *	0.416	0.125	0.011 *
Women	4.2 (2.7)	4.7 (2.9)	6.5 (3.5)	<0.0001 *	0.119	<0.0001 *	<0.0001 *
**Past month heaviest AMED Occasion**							
Men	7.1 (6.1)	9.7 (6.8)	12.5 (9.7)	<0.0001 *	<0.0001 *	0.111	<0.0001 *
Women	5.2 (4.4)	6.2 (5.2)	8.1 (6.3)	<0.0001 *	0.044 *	0.002 *	<0.0001 *

Mean and SD (between brackets) number of alcoholic drinks are shown. Post-hoc pairwise comparisons were conducted with Bonferroni’s correction. * = Differences are significant if *p* < 0.05. Abbreviations: AO = alcohol only; AMED = alcohol mixed with energy drink.

**Table 8:** Energy drinks mixed with alcohol consumption and risk-taking.

	Number of Energy Drinks on AMED Occasion			Overall	
RT-18 Risk Taking Group	Low (0–5)	Moderate (6–12)	High (13–18)	*p*-Value	Corrected *p*-Value ^1^
**Usual AMED occasion**					
Men	1.8 (1.3)	2.1 (1.8)	2.4 (2.3)	0.049 *	0.198
Women	1.8 (1.3)	1.6 (1.2)	1.9 (1.3)	0.046 *	0.077
**Past Month Heaviest AMED Occasion**					
Men	2.4 (1.6)	3.1 (2.6)	3.7 (3.2)	0.001 *	0.036 *
Women	2.3 (1.5)	2.2 (1.6)	2.6 (2.2)	0.025 *	0.148

Mean and SD (between brackets) number of energy drinks consumed on AMED occasions are shown. Post-hoc pairwise comparisons were conducted with Bonferroni’s correction. * = Differences are significant if *p* < 0.05. ^1^ = *p*-value corrected for the number of alcoholic drinks consumed. Abbreviations: AMED = alcohol mixed with energy drink.

**Table 9:** Alcohol consumption and negative alcohol-related consequences.

	Number of Alcoholic Drinks			Overall	Pairwise Comparisons		
BYAACQ Group	Low (0–4)	Moderate (5–8)	High (9–24)	*p*-Value	Low vs. Moderate	Moderate vs. High	Low vs. High
**Usual AO Occasion**							
Men	6.9 (4.4)	8.4 (5.3)	11.5 (6.4)	<0.0001 *	0.011 *	<0.0001 *	<0.0001 *
Women	4.6 (2.7)	6.1 (3.4)	8.0 (4.5)	<0.0001 *	<0.0001 *	<0.0001 *	<0.0001 *
**Past Month Heaviest AO Occasion**							
Men	11.4 (6.2)	14.9 (7.3)	18.7 (8.4)	<0.0001 *	<0.0001 *	<0.0001 *	<0.0001 *
Women	7.6 (4.5)	10.3 (5.2)	12.7 (6.3)	<0.0001 *	<0.0001 *	0.001 *	<0.0001 *
**Usual AMED Occasion**							
Men	6.1 (4.1)	8.8 (5.0)	8.3 (5.0)	<0.0001 *	<0.0001 *	1.000	<0.0001 *
Women	4.2 (2.6)	5.5 (3.3)	6.2 (3.7)	<0.0001 *	<0.0001 *	0.640	<0.0001 *
**Past Month Heaviest AMED Occasion**							
Men	7.7 (6.2)	12.4 (8.8)	11.3 (7.8)	<0.0001 *	<0.0001 *	1.000	<0.0001 *
Women	5.1 (4.1)	7.8 (6.4)	8.2 (6.2)	<0.0001 *	<0.0001 *	1.000	<0.0001 *

Mean and SD (between brackets) number of alcoholic drinks are shown. Post-hoc pairwise comparisons were conducted with Bonferroni’s correction. * = Differences are significant if *p* < 0.05. Abbreviations: BYAACQ = brief young adult alcohol consequences questionnaire, AO = alcohol only; AMED = alcohol mixed with energy drink.

**Table 10:** Energy drinks mixed with alcohol consumption and negative consequences.

	Number of Energy Drinks on AMED Occasion			Overall	Pairwise Comparisons			
BYAACQ Group	Low (0–4)	Moderate (5–8)	High (9–24)	*p*-Value	Low vs. Moderate	Moderate vs. High	Low vs. High	Corrected *p*-Value ^1^
**Usual AMED Occasion**								
Men	1.8 (1.4)	2.1 (2.0)	2.7 (2.2)	<0.0001 *	0.782	<0.0001 *	<0.0001 *	<0.0001 *
Women	1.5 (1.0)	2.1 (1.6)	2.0 (1.4)	<0.0001 *	<0.0001 *	1.000	0.001 *	<0.0001 *
**Past Month Heaviest AMED Occasion**								
Men	2.5 (2.1)	3.3 (3.1)	3.7 (2.8)	<0.0001 *	0.004 *	0.111	<0.0001 *	0.002 *
Women	2.0 (1.6)	2.7 (1.7)	2.7 (1.9)	<0.0001 *	<0.0001 *	1.000	<0.0001 *	0.005 *

Mean and SD (between brackets) number of energy drinks consumed on AMED occasions are shown. Post-hoc pairwise comparisons were conducted with Bonferroni’s correction. * = Differences are significant if *p* < 0.05. ^1^ = *p*-value corrected for the number of alcoholic drinks consumed. Abbreviations: BYAACQ = brief young adult alcohol consequences questionnaire; AMED = alcohol mixed with energy drink.

## Data Availability

The data is available on request from the corresponding author.
